# Biometrics and Biomarkers in Patients With Psoriasis

**DOI:** 10.7759/cureus.73929

**Published:** 2024-11-18

**Authors:** Sebastian Arango, Kawaiola Cael Aoki, Shakil O Huq, Alexander Blanca, Marc M Kesselman

**Affiliations:** 1 Medical School, Kiran C. Patel College of Osteopathic Medicine, Nova Southeastern University, Davie, USA; 2 Rheumatology, Kiran C. Patel College of Osteopathic Medicine, Nova Southeastern University, Davie, USA

**Keywords:** basophils, biometrics, dermatology, nhanes, psoriasis

## Abstract

Psoriasis (PsO) is a chronic, systemic, and autoimmune dermatologic condition characterized by dry, scaly, and erythematous plaques on the skin. PsO can present in various forms, including guttate (small, round lesions commonly over the upper trunk and extremities that can be raised and scaly), inverse (smooth plaques of inflamed skin within skin folds of the groin, buttock, and breasts), pustular (white painful pustules within red inflamed blotches widespread over the body), and erythrodermic (red rash present over most of the body). Individuals with PsO can present differently, with unique symptoms and patterns on the skin. These diverse manifestations make PsO a complex condition with mild to severe symptoms affecting different body areas. Researchers have identified intrinsic risk factors (and comorbidities) tied to PsO, including genetics, obesity, metabolic syndrome, infection, cardiovascular disease, stress, and type 2 diabetes mellitus (T2DM). In addition, several extrinsic risk factors have also been shown to be tied to PsO onset and progression, such as ultraviolet (UV) light, air pollution, and various pharmacological treatments.

While these intrinsic and extrinsic factors have been tied to disease pathophysiology, the underlying mechanisms of disease activity have yet to be elucidated fully, making diagnosis and treatment cumbersome. Currently, PsO is diagnosed clinically with no definitive test. Noninvasive tools such as dermoscopy aid in diagnosis, while the biopsy is reserved for difficult-to-characterize psoriatic-like lesions. The reliance on clinical presentation and the lack of diagnostic testing available have led to the underdiagnosis of PsO, particularly in minority communities. The goal of this study is to utilize data from the National Health and Nutrition Examination Survey (NHANES) to improve the diagnosis of PsO and target treatment more effectively, and biometric measurements associated with PsO should be studied to aid clinical practitioners in better understanding the disease pathophysiology and improve patient diagnosis, management, and prognosis. Using the dataset, we conducted a retrospective cohort study to find which variables are significantly associated with PsO. These objective measurements can complement clinical assessments by providing quantifiable data that could improve accuracy by detecting PsO in its early stages or distinguishing it from other skin conditions with similar presentations. This enables healthcare providers to adjust management strategies based on measurable changes in disease markers, rather than relying solely on subjective clinical observations.

## Introduction

Psoriasis (PsO) is a chronic, systemic, and autoimmune dermatologic condition affecting the skin, hair, nails, and joints. PsO can present in various forms, with the most common being chronic plaque PsO, which appears as symmetrical, well-defined papules or plaques with silvery-white scales. Other, less common variants include guttate PsO (small, uniformly round plaques or pinpoint lesions commonly found on the upper trunk and extremities that can be raised and scaly), inverse PsO (smooth plaques of inflamed skin within skin folds, such as the groin, the buttocks, and under the breasts), pustular PsO (sterile pustules on an erythematous base that can coalesce widely over the body), and erythrodermic PsO (generalized erythema covering most of the body). Individuals with PsO can present differently, with unique symptoms and patterns on the skin. The classic presentation is described as erythematous plaques with silvery scales on extensor surfaces, such as the skin, hair, or nails, and varies depending on location and lesion morphology [1]. PsO is commonly chronic, with recurrent exacerbations and remissions, which can be triggered or worsened by various factors.

Approximately eight million US adults are living with this chronic condition, which is believed to be underreported, especially among minority populations, and is also expected to grow with the aging population [2,3]. The prevalence of PsO is estimated to be approximately 2-3% within the United States, which is similar among men and women but more prevalent among Caucasian individuals as compared with Hispanics, African Americans, and other non-White individuals. Research efforts and clinical visits for PsO have lagged among minority communities, which has resulted in a disproportionate amount of the disease burden and severity and poorer prognosis among these populations [4,5].

The pathophysiology of PsO has become more apparent over the next decade, although there is still much to be learned. The onset and progression of PsO, associated with the dysregulation of the immune system, result in inflammation mediated by lymphocytes, causing epidermal hyperplasia (also referred to as acanthosis) [6]. The exact etiology associated with PsO remains unclear. Still, research has identified genetic predispositions and environmental triggers that induce damage to keratinocytes, initiating a sequence of transcriptional events that eventually result in plaque formation [7,8]. Vascular endothelial growth factor (VEGF) has been implicated in the initiation and progression of PsO, leading to its characteristic histopathological findings [9]. The chronic inflammatory process is maintained by the innate, adaptive, and resident immune cells within the skin. The condition commonly occurs in two stages: the initiation phase and the maintenance phase. It is widely accepted that dendritic cells (DCs) play a central role in initiating the cascade of cellular events, ultimately leading to PsO's pathology. The activation of DCs, antigen-presenting cells (APCs), remains unclear. Still, current research findings suggest that proteins secreted by keratinocytes, due to either genetic susceptibility or local trauma/inflammation, lead to their activation and maturation [10].

The diagnosis of PsO primarily relies on clinical evaluation, focusing on cutaneous and/or arthritic symptoms, supported by changes observed in dermoscopy and biopsy. This approach can be challenging for clinicians due to the broad range of differential diagnoses and varying experience levels in treating patients with diverse skin tones. No established biometric or unique biosignature has been widely accepted, but specific correlated values can provide a more informed perspective.

Treatment commonly varies by type of PsO and severity of disease. Specifically, topical steroids are widely used for individuals with mild symptoms, while systemic medications that inhibit cell division or biologic therapies that target inflammatory cytokines, including tumor necrosis factor-alpha (TNF-α), interleukin-17 (IL-17), and interleukin-23 (IL-23) inhibitors, are commonly used for a more moderate to severe disease presentation [11,12]. PsO also has many extra-cutaneous effects, which can lead to a dramatic decline in quality of life. These effects, along with the increased incidence of comorbidities such as cardiovascular disease and mental health disorders, are tied to PsO [13,14]. Organizations, such as the World Health Organization (WHO) and the National Psoriasis Foundation, have emphasized the need for the rapid diagnosis and management of PsO to effectively reduce the burden of PsO on the healthcare systems and the economy at large.

This study uses the most recent National Health and Nutrition Examination Survey (NHANES) data to re-evaluate the biometric measurements and modifiable risk factors associated with PsO. With a better understanding of these cofactors, which clinical practitioners could target, and a better understanding of disease pathophysiology, clinicians can improve PsO diagnosis and management. Addressing this unmet need is essential in reducing PsO's disease burden on the healthcare system and economy while also addressing disparities in management.

## Materials and methods

Study design and population

A statistical analysis examined the association between PsO and various biometrics, including anthropometric measurements, inflammatory proteins, white blood cell differentials, minerals, and vitamin levels. Data was collected from baseline interviews and health examinations using the NHANES, a national program designed to assess the health and nutritional status of a nationally representative sample of the resident, civilian, non-institutionalized US population biennially. The analysis utilized NHANES data between 2013 and 2014 from participants who completed a series of interview questionnaires through a computer-assisted personal interviewing system, followed by a standardized examination in a mobile examination center. These interviews included questions related to health-related risks, attitudes, and behaviors. To better understand the health of specific sub-populations, the survey employs oversampling strategies for Hispanic individuals, non-Hispanic Black individuals, low-income White individuals (≤130% of the federal poverty level), and adults over 80. This dataset was leveraged to analyze various variables, covering demographic details, physical examination findings, laboratory results, and questionnaire outcomes. Demographic factors examined included age, with individuals aged 80 years and older represented as 80, gender, and ethnicity, categorized into Mexican American, other Hispanic, non-Hispanic White, non-Hispanic Black, non-Hispanic Asian, and other race or multi-racial groups. Additional demographic information such as income, education level, and marital status were also considered. Physical examinations were conducted in mobile examination centers by trained health technicians to ensure consistency and minimize variability. Anthropometric measurements followed standardized protocols. Height was measured with participants standing without shoes, ensuring their heels, buttocks, and back touched the vertical bar, with heads positioned in the Frankfort plane. Weight was collected using a digital scale while participants wore light clothing and no shoes. Waist circumference was measured at the top of the iliac crest using a flexible tape measure, with participants standing upright. Lastly, sagittal abdominal diameter was measured using a caliper or similar tool, with participants lying supine. Additionally, laboratory evaluations included analyses of complete blood counts (CBCs), vitamins, minerals, carotenoids, albumin, calcium, and cholesterol. All persons in supervised care or custody in institutional settings, active-duty military personnel, active-duty family members living overseas, and any other US citizens residing outside the 50 states and the District of Columbia were excluded from the NHANES dataset.

Statistical analysis

Individuals included in the study encompassed all non-pregnant adults over 18 years of age who were not in supervised care or custody in institutional settings, active military personnel, active-duty family members living outside of the United States, or any other citizens residing outside the 50 states, the District of Columbia, and its associated territories. The analysis included a total sample size of 12,625 participants who had valid data on PsO. Statistical analysis was conducted using IBM SPSS Statistics for Macintosh, Version 29.0.0.0 (Released 2019; IBM Corp., Armonk, New York, United States). The independent predictors were derived from the NHANES Laboratory Data and treated as continuous variables. The primary outcome, a history of PsO, was determined based on responses to the medical questionnaire and coded as a binary variable, specifically through the question "Were you ever told that you had psoriasis (MCQ070)?" indicating a physician's previous diagnosis. Covariates of interest in the analysis included demographic factors such as age, gender, ethnicity, and anthropometric measures such as body mass index (BMI), waist circumferences, and sagittal abdominal diameter. Responses categorized as "no response" were re-coded as "NA" and analyzed accordingly. In addition, individuals with a "weak positive" result for tissue transglutaminase IgA (tTG-IgA) were re-classified as "positive" in the analysis. An α prior significance level was set at 0.05 for the study.

Demographic data analysis was conducted utilizing t-tests and chi-squared tests. Univariable logistic regression analysis was performed for all predictors. In the multivariable model, predictors with a p-value of 0.25 or lower were considered for inclusion. Non-significant potential confounders were systematically removed stepwise to establish the final multivariable model. The analysis revealed no significant interactions.

## Results

Significant results from the univariable analysis are shown in Table 1. tTg-IgA, neutrophil percentage, percentage age, sagittal abdominal diameter, waist circumference, and neutrophil number were all statistically significant for being associated with PsO, while lymphocyte percentage and basophil number were protective and associated with a decreased risk of PsO. A weak positive/positive qualitative test to tTg-IgA increased the odds of having PsO by approximately 4.5 times (OR=4.49 (1.91-10.54)). The CBC data, neutrophil percentage, and neutrophil number (1000 cells/uL) increased odds by 3.2% and 9.3% per unit increase, respectively. For anthropometric measures, waist circumference and sagittal abdominal diameter increased the odds of having PsO by 1.4% and 4.6% per centimeter, respectively. Percentage age (years) was associated with increased odds of developing PsO (OR=1.02 (1.01-1.03)). Lymphocyte percentage (OR=0.96 (0.94-0.97)) demonstrated a protected effect.

**Table 1:** Significant results from the univariable analysis Significant p-values: <0.05*, <0.01**, <0.001*** OR: odds ratio; CI: confidence interval

Variable	OR	95% CI	P-value
Tissue transglutaminase IgA	4.486	1.910-10.538	<0.001***
Neutrophil percentage	1.032	1.020-1.045	<0.001***
Percentage age (years)	1.02	1.01-1.03	<0.001***
Sagittal abdominal diameter	1.046	1.020-1.073	<0.001***
Waist circumference	1.014	1.007-1.020	<0.001***
Lymphocyte percentage	0.957	0.944-0.971	<0.001***
Neutrophil number	1.093	1.031-1.158	0.003**
Creatine phosphokinase	0.999	0.997-1.000	0.011*

Results from the multivariable logistic regression are shown in Table 2. Significant factors included age (p=0.005), tTg-IgA (p=0.003), lymphocyte percentage (p=0.008), and basophil number (p=0.035). This analysis revealed that tTg-IgA nearly pentupled the odds of having PsO (OR=4.99 (1.735-14.35)). Age also increased the odds of having PsO by 1.2% per year. Lymphocyte percentage (OR=0.97 (0.96-0.99)) and basophil number (OR=0.032 (0.001-0.78)) demonstrated a protective effect.

**Table 2:** Results from the multivariable logistic regression Significant p-values: <0.05*, <0.01**, <0.001*** OR: odds ratio; CI: confidence interval

	OR	95% CI	P-value
Constant	0.037		<0.001***
Tissue transglutaminase IgA	4.99	1.74-14.35	0.003**
Age (years)	1.012	1.004-1.020	0.005**
Lymphocyte percent (%)	0.974	0.96-0.99	0.008*
Basophil number (1000 cell/uL)	0.032	0.001-0.781	0.035*

## Discussion

tTG-IgA

PsO is a chronic disease with a multifactorial etiology associated with inflammation. The pathogenesis is believed to be due to plasmacytoid dendritic cells (pDCs) residing in the skin and secreting interferon (IFN), which results in myeloid DCs secreting large quantities of TNF-α, IL-23, and IL-12, leading to an inappropriate Th1, Th17, and Th22 immune response [7,15,16]. Once APCs activate T cells, they release cytokines to recruit leukocytes and lymphocytes, such as neutrophils and B cells, which migrate to the skin and are considered the hallmark indicator of psoriatic pathogenesis [13]. PsO is maintained and progresses through the activation of T cells, which gain memory and perpetuate the chronic nature of the disease, as they secrete inflammatory cytokines, even in those with no active lesions [17,18]. The dominant cytokine axis is IL-17/IL-23. IL-17 is secreted by Th17 cells in response to IL-23, creating a positive feedback loop, with these being found high in psoriatic lesions [17,19]. IL-17 is crucial in promoting pro-inflammatory pathways such as NF-κB and MAPK signaling pathways, whereas IL-23 supports the differentiation of T cells into Th17 [19]. The condition can be exacerbated by various comorbidities, including metabolic syndrome, obesity, and type 2 diabetes mellitus (T2DM), which also increase the risk of mortality [13]. Autoimmune conditions, including Crohn's disease and ulcerative colitis, are more likely to occur among individuals with PsO [20]. The activation of Th1 and Th17 T cells secondary to autoimmune diseases likely predisposes an individual to other autoimmune manifestations because of the hypersecretion of cytokines such as TNF-α, IL-23, and IL-17, which is discussed in greater detail below.

The efficacy of immune modulators such as TNF-α inhibitors, Janus kinase (JAK) inhibitors, and IL-12/IL-23 inhibitors, which target underlying autoimmune disease activity, and the associated flares tied to PsO that occur due to molecular mimicry following infections suggest an autoimmune pathophysiology [10]. The association of PsO and other autoimmune diseases continues to be fully elucidated, but we provide insight into some potential pathways outlined in recent research efforts. Over the last 25 years, an association between tTg-IgA and PsO has evolved. While tTg-IgA has been more closely associated with celiac disease, another autoimmune condition, our findings support an existing body of literature regarding tTg-IgA and its potential overlap with the pathophysiology of PsO [21]. One study found that patients with celiac disease who tested positive for tTg-IgA had significantly higher levels of pro-inflammatory cytokines, including IFN-γ, TNF-α, and IL-6, compared to patients without tTg-IgA [22]. This finding suggests that the inflammatory cytokines seen in celiac disease responsible for producing tTg-IgA are related to the inflammatory cascade causing psoriatic symptoms, as elevations in IFN-γ, TNF-α, and IL-6 are also seen in PsO [7,15,16,23]. While the association remains poorly understood, this intersection in pathophysiology suggests a shared commonality of inflammatory autoimmune dysregulations requiring further research.

Of the biometrics we identified as statistically significant as part of the NHANES data, tTg-IgA demonstrated the strongest association with PsO, with an OR of 4.486. This means that a quantitative positive test for tTg-IgA increases the odds of having PsO by almost five times. This association could bring attention to patients who may be undiagnosed or experiencing sensitivity, allowing for potentially other avenues of treatment that are multifaceted and target possible comorbidities. Specifically, tTg-IgA testing may be particularly valuable in patients with PsO who have comorbid autoimmune diseases, unexplained gastrointestinal symptoms, recurrent or refractory flare-ups, a strong family history of autoimmune conditions, or concurrent metabolic syndrome or T2DM.

We reviewed six studies that examined the relationship between tTg-IgA and PsO. One showed a significantly higher antibody ratio in patients with PsO than those without the condition. Interestingly, the same study revealed that women with PsO were more likely to have the presence of tTg-IgA than their male counterparts. In addition, elderly patients with PsO were more likely to have the antibody present than their younger peers [24]. Another study reported that patients with PsO have significantly higher concentrations of tTg-IgA than patients without the condition. They also found that higher levels of the antibody correlate with higher PsO activity measured using the Psoriasis Area and Severity Index (PASI) [21]. Another report supported this finding, which found that higher levels of celiac disease antibodies, including tTg-IgA, were associated with disease severity, measured by the current or previous use of systemic immunosuppressants for their PsO [25].

Meanwhile, a conflicting body of literature exists, which fails to identify a significant association between tTg-IgA alone and PsO [21,25-27]. While two studies found elevated levels of tTg-IgA in psoriatic patients compared to those without, they were not statistically significant [25,26]. Two of the six articles found no association with PsO and tTg-IgA [27,28]. Additional research is needed to understand better the relationship between tTg-IgA presence/concentration and PsO. A focus on improving the power of future studies with larger sample sizes should be had, as no study on the topic had more than 65 psoriatic patients, which may influence current data.

Another signal transduction pathway being explored for its association with PsO is the IL-23/IL-17 cytokine pathway. In this pathway, a self-amplifying cascade is ignited when IL-23, produced mainly by DCs, stimulates lymphocyte differentiation into Th17 cells, which produce IL-17. This causes keratinocyte proliferation and infiltration by other inflammatory cells, particularly neutrophils. Inhibition of IL-17 and IL-23 has been influential in treating patients with moderate to severe PsO [29]. IL-6 is another interleukin associated with PsO, which may play a role in the disease pathophysiology via the IL-23/IL-17 pathway. IL-6 also induces keratinocyte proliferation and the acute phase response and is significantly higher in the serum of patients with PsO than the serum of patients without PsO, albeit not correlating with severity [30,31]. Other inflammatory cytokines, including IL-22, IFN-γ, and TNF-α, have also been associated with PsO due to their pro-inflammatory effects on keratinocytes. One study found that IL-22 was increased in the serum of patients with PsO and had an anti-apoptotic effect on keratinocytes, likely attributable to the upregulation of Bcl, an anti-apoptotic protein, and downregulation of Bax, a pro-apoptotic protein [32]. Another study reported significantly increased serum IFN-γ, which would induce dendritic cells to produce IL-23, potentiating the IL-23/IL-17 pathway [33]. A further study found that TNF-α was significantly higher in the serum of patients with PsO as compared to patients without the condition but did not correlate to disease severity [34].

Leukocytes and lymphocytes

The relationship between PsO and certain leukocytes, particularly the role of basophils, still needs to be fully elucidated, with limited presence in the literature. Meanwhile, the involvement of lymphocytes, such as neutrophils, is well-documented in the pathogenesis of PsO and serves as a target for numerous treatments [35]. The univariable analysis conducted in this study of NHANES data demonstrated similar relationships between what has been found in multiple studies, including extensive population studies showing that neutrophils are predictive and associated with an increased risk of PsO [35-38]. Recent evidence points to the neutrophil-to-lymphocyte ratio being a possible helpful biometric [39,40]. Neutrophils are the primary infiltrate present among patients with PsO, playing a crucial role in perpetuating inflammation by secreting cytokines such as TNF-α, IL-4, IL-8, IL-17, and IL-23, which further recruit lymphocytes [41]. Still, study results have been mixed regarding whether the presence or absence of basophils indicates disease etiology.

Basophils play a role in Th2 immune responses and are responsible for atopic reactions that increase IgE. This activates eosinophils and recruits mast cells to release histamine. Allergic reactions have been established in other inflammatory skin conditions, such as atopic dermatitis (AD), but these reactions do not play a key role in PsO onset and progression [36]. The mechanisms of disease activity tied to various inflammatory skin diseases, including AD, hidradenitis suppurativa, and alopecia areata, are associated with cytokine pathways that play a role in disease onset and progression by mediating the activation and recruitment of immune cells. Exploring the difference between the immune response in PsO and other inflammatory skin conditions, including AD, can assist in distinguishing between the two conditions. Patients with PsO were not found to have basophilic infiltration as with AD and other inflammatory skin diseases [42,43]. This supports the statistically significant association of basophils with PsO and their protective properties [43]. Pathogenesis of PsO appears to differ from allergic conditions, which provides a likely explanation as to why our study found basophils to be protective of PsO due to their presence being indicative of an atopic process, mediated by Th2, IgE, and basophils [44].

Creatine phosphokinase

Creatine phosphokinase (also known as creatine kinase (CK)) is a muscle enzyme critical for cellular energy production, especially during high energy demand or ischemia. The role of CK in the brain, skeletal muscle, and cardiac muscle has been well-established, making it a valuable serologic diagnostic tool for clinicians. Meanwhile, its role in PsO remains speculative and needs to be fully understood. Some case studies have shown patients with psoriatic arthritis presenting with elevated levels of CK, hinting at a potential link that requires further investigation [45,46]. CK helps to support normal cellular function but may be involved in healing in hyperproliferative diseases due to the upregulation of cellular synthesis [47]. The speculated connection between PsO and CK may stem from its association with dermatomyositis (DM), an autoimmune condition that, like PsO, relies on the IL-17/IL-23 cytokine pathway for its pathogenesis [10,48]. In DM, elevated CK levels result from myocyte damage caused by inflammation, leading to the release of this enzyme into the bloodstream. The precise relationship between PsO and CK, particularly in their potential link to DM, remains unconfirmed and novel, meriting additional research to understand better how these diseases are interconnected.

Sagittal abdominal diameter and waist circumference

The sagittal abdominal diameter and waist circumference are anthropometric measurements used to assess abdominal obesity, a critical component of metabolic syndrome. These measurements indicate visceral fat accumulation, a significant risk factor for cardiovascular diseases, insulin resistance, and systemic inflammation, suggesting that obese patients with PsO have diets that consist of more saturated fats and alcohol [49]. It is hypothesized that the dysregulation caused by having more adipose tissue can allow adipocytes to secrete inflammatory mediators like leptin and resistin that directly lead to a pro-inflammatory profile that not only increases insulin resistance but also induces the production of Th17, which is mainly responsible for the pathogenesis of many autoimmune diseases [50]. Th17 being more abundant may significantly explain why PsO is linked with obesity and why these anthropometric measurements are essential [51].

Furthermore, the inflammatory pathways activated in PsO, including increased TNF-α, IL-6, and other pro-inflammatory cytokines, are also implicated in the pathogenesis of obesity and metabolic syndrome. Specifically, it has been found that the cytokine signaling from the TNF family within the adipose tissue is responsible for attracting inflammatory macrophages that lead to liver steatosis and insulin resistance. The increased number of dead adipocytes in the obese population furthers a state of lipotoxicity that creates an environment of more pro-inflammatory cytokines that allow hepatocytes to be killed. In these ways, inflammatory responses change metabolic processes like insulin signaling and energy homeostasis [52]. These findings strengthen the link where higher sagittal abdominal diameter and waist circumference, as indicators of abdominal obesity, could contribute to the systemic inflammation seen in PsO, potentially worsening the disease's severity.

Moreover, weight loss has been shown to improve PsO severity and treatment outcomes. Obese individuals with PsO exercise less on average than obese individuals without PsO [51]. This suggests that interventions targeting reductions in sagittal abdominal diameter and waist circumference might decrease the risk of metabolic syndrome and ameliorate the systemic inflammation associated with PsO, leading to better disease management and quality of life for affected individuals.

Age (years)

The relationship between increasing age and PsO is multifaceted, with implications for the disease's onset, progression, and management. PsO can manifest at any age but is often seen within two peak periods of onset, including one in the second decade of life and the other in the fifth and sixth decades [53]. This suggests a complex interplay between genetic predisposition, environmental factors, and age-related changes in immune system function. As individuals age, several factors can influence the expression and severity of PsO. First, the immune system undergoes a process of senescence, which can alter the body's inflammatory response. While the overall immune response may decrease with age, the inflammatory aspect of the immune system can become more active, potentially exacerbating chronic inflammatory conditions like PsO. This heightened inflammation in older adults could worsen PsO symptoms or lead to more persistent plaques. In addition, the skin itself changes with age, becoming thinner, less elastic, and more prone to dryness and damage. These age-related changes in skin barrier function can influence the severity of PsO plaques and the effectiveness of topical treatments. Elderly patients may also have a different response to PsO therapies due to alterations in drug metabolism associated with aging, changes in their microbiome, and the increased likelihood of comorbid conditions requiring medication, raising the risk of drug interactions and side effects [54]. For example, it has been found that gut microbiome changes among elderly individuals are associated with *Prevotella* abundance, which can be linked with increased Th17 and the exacerbation of psoriasiform skin inflammation. Their experiment tested fecal implants in mouse models, which reduced the abundance of *Prevotella *found in their colon and alleviated psoriatic-like symptoms [55]. In summary, increasing age can affect the onset, progression, and treatment of PsO through changes in immune function, skin physiology, gut microbiome, and comorbid conditions. These factors underscore the need for age-specific approaches to managing and caring for PsO, carefully considering the broader implications of aging on disease activity and patient well-being.

Limitations

Certain limitations are inherent to this study due to the nature of the NHANES data. As an observational, cross-sectional dataset, NHANES is designed to establish associations rather than causative relationships, offering valuable insights but not direct causation. Using retrospective NHANES data provides a robust basis for analysis but may limit control over certain confounding factors or ensure the inclusion of all variables directly related to PsO. The statistical methods employed, such as multivariable analyses, effectively control for variables available in the model, although unmeasured factors may still contribute. NHANES data primarily represent the American population and those who self-reported PsO, providing meaningful information on this demographic; however, findings may not fully extend to more diverse global populations or those with different severities of PsO. Differences in demographics, such as age, ethnicity, and gender, also suggest that findings may vary in relevance across other groups with different risk profiles.

Currently, no serologic diagnostic tests or laboratory markers are established for PsO, so the study relies on clinical data that, while informative, may lack full diagnostic precision. Biomarkers used in this analysis, such as neutrophil levels, lymphocyte percentages, and CK levels, may vary due to factors unrelated to PsO (e.g., infections, medications, stress), which can impact their specificity for the condition. Although not included in this study, other potential biomarkers and lifestyle factors (diet, stress, etc.) could further inform PsO risk and progression, meriting future research to expand these findings.

## Conclusions

PsO is a chronic inflammatory skin disease with a relapsing and remitting course diagnosed mainly on clinical observations, as there are currently no established serologic diagnostic tests or laboratory markers. The study aimed to find specific biometrics associated with psoriatic patients and better understand them, shedding light on their risk factors and comorbidities. Currently, many biomarkers such as IL-6, IL-10, IL-1, IL-2, IL-17, IL-22, IL-33, IFN-γ, TNF-α, TARC, lipocalin-2, resistin, adiponectin, leptin, and calprotectin are being studied. After analyzing the most recent NHANES data, several significant parameters were discovered. Notably, using multivariable analysis, age at screening and tTG-IgA emerged as the most positively associated with PsO. An elevated tTG-IgA level indicated a roughly fivefold increased risk of PsO. In addition, lymphocyte percentage and number of basophils showed a decreased association, suggesting their potential role in developing a qualitative biometric profile indicating a lower risk of PsO, though not diagnostic. While neutrophils' significant role in PsO is well-established, our study highlighted the possible differentiating disease etiology of basophils, an area relatively unexplored in the existing literature. In addition, individuals with greater abdominal diameter and waist circumference were correlated with a higher risk of PsO due to its relationship with metabolic syndrome and chronic inflammation associated with increased visceral fat. Psoriatic patients often experience extensive disease burden and multiple comorbidities, posing potential life-threatening risks. This study identified CK as a significant marker in PsO, albeit with the weakest association. While no direct psoriatic pathophysiology results in creatine phosphokinase elevation, case studies suggest a rare link between psoriatic arthritis and DM, providing a potential explanation for its significance in our research that requires further elucidation. This opens new avenues for researchers to explore. Future studies should include a quantitative dataset on tTG-IgA to clarify specific serological biomarkers or biometric values associated with an increased risk of PsO, potentially offering diagnostic insights as this study found correlations with PsO. Ultimately, when determining a diagnosis for patients with PsO, their comorbidities may demonstrate influences that may be exacerbating their symptoms and offer other avenues of controlling and identifying the drivers of their disease.
